# Reliability of core needle biopsy for HER2-low early-stage breast cancer

**DOI:** 10.1007/s12094-025-03877-2

**Published:** 2025-03-09

**Authors:** C. M. Ciniselli, P. Verderio, V. Duroni, P. Baili, S. Pizzamiglio, F. G. de Braud, S. Folli, C. Depretto, G. Scaperrotta, M. C. De Santis, M. G. Carnevale, C. De Marco, A. Vingiani, G. Pruneri, S. Di Cosimo

**Affiliations:** 1 Bioinformatics and Biostatistics Unit, Department of Epidemiology and Data Science, Fondazione IRCCS Istituto Nazionale dei Tumori, 20133 Milan, Italy; 2 Data Science Unit, Department of Epidemiology and Data Science, Fondazione IRCCS Istituto Nazionale dei Tumori, 20133 Milan, Italy; 3 Department of Medical Oncology and Hematology, Fondazione IRCCS Istituto Nazionale dei Tumori, 20133 Milan, Italy; 4 University of Milan, Milan, Italy; 5 Breast Unit, Fondazione IRCCS Istituto Nazionale dei Tumori, 20133 Milan, Italy; 6 Breast Imaging Unit, Fondazione IRCCS Istituto Nazionale dei Tumori, 20133 Milan, Italy; 7 Radiation Oncology 1, Fondazione IRCCS Istituto Nazionale dei Tumori, 20133 Milan, Italy; 8 Department of Advanced Diagnostics, Fondazione IRCCS Istituto Nazionale dei Tumori, 20133 Milan, Italy

**Keywords:** HER2-low, Early-stage breast cancer, Concordance, Immunohistochemistry, Core needle biopsy, Surgical samples

## Abstract

**Background:**

The reliability of core needle biopsy (CNB) for HER2-positive breast cancer is well established. However, data on HER2-low and the potential for inconsistencies with surgical samples are limited.

**Materials and methods:**

Concordance between CNB and surgical samples was assessed using the unweighted Cohen kappa statistic (Kc) in a consecutive series of 776 treatment-naïve early-stage breast cancer patients. Logistic regression models were used to evaluate the association between concordance and clinico-pathological features.

**Results:**

The agreement for HER2-positive status between CNB and surgical specimens was high at 95%, with a Kc value of 0.86 indicating almost perfect agreement. However, 65 of 123 (53%) cases initially classified as HER2-0 were reclassified as HER2 1 + or 2 + /ISH-negative, and 89 of 374 (24%) cases initially classified as HER2 1 + /2 + were HER2-0 in surgical samples. This resulted in a Kc value of 0.22, indicating fair agreement in classifying HER2-0 versus HER2-low breast cancer. Tumor size was a significant factor influencing discordance, with tumors larger than 2 cm having double the risk of misclassification.

**Conclusion:**

These findings suggest that HER2 status should be retested, particularly for large tumors initially diagnosed as HER2-0, in light of new effective therapies for HER2-low breast cancer, such as antibody–drug conjugates.

**Supplementary Information:**

The online version contains supplementary material available at 10.1007/s12094-025-03877-2.

## Introduction

Assessment of HER2 status is crucial in breast cancer diagnostics. While surgical findings are considered the gold standard, the evaluation of core needle biopsy (CNB) is preferred due to its accessibility, cost-effectiveness, and convenience, especially for patients undergoing neoadjuvant therapy [1–3]. CNB has been shown to be reliable, and anti-HER2 treatment is given based on CNB results for patients with HER2-overexpressing/amplified (HER2-positive) tumors [4]. The development of novel antibody–drug conjugates (ADCs) has advanced targeted therapy beyond HER2-positive breast cancer. Clinical trials with trastuzumab deruxtecan (T-Dxd, DS-8201) have shown impressive results in metastatic HER2-low breast cancer, defined by 1 + and 2 + immunohistochemistry (IHC) scores without HER2 gene amplification [5]. This success encourages further exploration of new ADCs and supports their use in earlier stages, including the neoadjuvant setting [6, 7]. Thus, accurately determining HER2 status is becoming more important. However, assessing HER2-low status may pose challenges with traditional methods such as IHC, which may have limitations in distinguishing between 0 and 1 + cases [8, 9]. Additionally, previous studies on the concordance between initial CNB and surgical specimens overlooked HER2 non-overexpressing cases due to their poor clinical relevance at the time. Therefore, the reliability of using preoperative CNB as the standard for diagnosing and planning treatment for HER2-low breast cancer, particularly with the potential introduction of ADCs for newly diagnosed cases, remains uncertain. The current study leveraged large prospectively collected data from a national cancer center to evaluate HER2-low status in both CNB and surgical specimens, focusing on factors that might affect the consistency of results.

## Patients and methods

Data for early-stage, treatment-naïve, breast cancer patients were collected from the prospectively maintained registry of Fondazione IRCCS Istituto Nazionale dei Tumori in Milan, Italy. The study focused specifically on consecutive patients with operable breast cancer who had HER2 immunohistochemistry (IHC) performed on both core needle biopsy (CNB) and surgical samples at the Institutional Department of Pathology between 2011 and 2022. The analysis was based on IHC data retrieved from the hospital breast cancer registry, and case revision was not performed. However, all cases were evaluated at our Institute by the same dedicated breast pathologists during the entire study period. HER2 was determined by IHC scoring from 0 to 3 + and by in situ hybridization (ISH), according to ASCO/CAP guidelines in place at the time of diagnosis [10]. HER2 status was considered as positive in case of IHC score 3 + or 2 + with amplified ISH, and HER2-negative otherwise, i.e., IHC 0, 1 + and 2 + without ISH amplification (ISH-negative). HER2-low were IHC 1 + or 2 + /ISH-negative. Estrogen receptor (ER) and progesterone receptor (PR) were labeled as negative (< 1%) or positive (≥ 1%), and Ki-67 as negative (< 20%) or positive (≥ 20%); hormone receptor (HR) status was classified as positive if either ER or PR was positive, or negative if both ER and PR receptors were negative. Approval for this study was granted by the Institutional Ethics Committee (INT 196-14). All patients signed the main study informed consent form, which included a non-specific clause for using collected patient and primary tumor data for future research.

### Statistical analysis

Standard descriptive statistics, median values and ranges for continuous variables and frequency tables for categorical variables were used to describe the main characteristics of the study population. The concordance between CNB and surgical samples was analyzed by computing the unweighted Cohen kappa statistics (Kc) [11] and its corresponding 95% confidence Interval (95%CI), and interpreted in a qualitative manner according to the Landis and Koch’s classification criteria [12]. The Sankey diagram was used to represent the changes among HER2-negative breast cancer, and univariate logistic regression models to evaluate the association between concordance and clinico-pathological characteristics [13]. All statistical analyses were done by the SAS Studio (version 5.2; SAS Institute, Inc., Cary, NC, USA), adopting a significance level of *α* = 0.05, graphical representations using the R software (R Foundation for Statistical Computing, Vienna, Austria) with the ggplot2 and forest plot packages.

## Results

A total of 7539 patient’s data were retrieved, and among these, 776 (10%) had paired HER2 IHC evaluations on both CNB and surgical specimens, and thus were included in the current study (Supplementary Fig. 1). Table 1 summarizes the clinico-pathological characteristics of the study population. The median age was 63 years (range: 25–93); 360 patients (46%) were post-menopausal, and 403 (52%) had a BMI < 25 kg/m^2^. Invasive ductal carcinoma was predominant. Nearly half of the patients had pT1 (51%) well/moderately differentiated (58%) breast cancer.

**Table 1:** Patient and primary tumor characteristics according to CNB and surgical specimens

Characteristics	*n *(%)
**Age (median, range)**	63, 25–93
< 50	190 (24.48)
≥ 50	586 (75.52)
**BM﻿I, kg/m**^**2**^	
< 25	403 (51.93)
≥ 25	343 (44.20)
*Missing*	*30 (3.87)*
**Menopausal status**	
Pre/Peri	200 (25.77)
Post	360 (46.39)
*Missing*	*216 (27.84)*
**Type of surgery**	
BCS	358 (46.13)
Mastectomy	415 (53.48)
*Missing*	*3 (0.39)*
**Pathology**	
Ductal	646 (83.25)
Lobular	109 (14.05)
Other	21 (2.71)
**Grade**	
I + II	451 (58.12)
III	318 (40.98)
*Missing*	*7 (0.90)*
**LVI**	
Present	245 (31.57)
Absent	388 (50.00)
*Missing*	*143 (18.43)*

	CNB	Surgical specimen
**Tumor size**		
≤ 2 cm	241 (31.06)	395 (50.90)
> 2 cm	335 (43.17)	364 (46.91)
*Missing*	*200 (25.77)*	*17 (2.19)*
**Nodal status**		
Negative	468 (60.31)	402 (51.80)
Positive	149 (19.20)	337 (43.43)
*Missing*	*159 (20.49)*	*37 (4.77)*
**Origin**		
Internal	776 (100)	776 (100)
Outside review	0 (0)	0 (0)

*BCS* breast-conserving surgery, *BMI* body mass index, *CNB* core needle biopsy, *LVI* lymphovascular invasion

Most of the cases (87%) were ER-positive at diagnosis. Observed agreement for ER status between CNB and surgical specimens was 98.18%, with a Kc of 0.92 (95%CI: 0.88–0.96), indicating almost perfect agreement (Table 2). The observed agreement for HER2 status, when categorized dichotomously as HER2-positive or -negative was 95.03%, with a Kc of 0.86 (95%CI: 0.79–0.92) indicating almost perfect agreement (Table 2). These findings were consistent over time, with a moderate to almost perfect level of agreement observed across the years of diagnosis (Supplementary Fig. 2).

**Table 2:** Concordance between CNB and surgical specimens

Biopsy	Surgery		Observed agreement	Concordance	
	Positive	Negative		Kc	95%CI
**ER**					
Positive	662	7	98.18%	0.92	0.88–0.96
Negative	7	94			
**PR**					
Positive	546	24	90.29%	0.73	0.67–0.79
Negative	50	142			
**HER2**					
Positive	67	11	95.03%	0.86	0.79–0.92
Negative	8	497			
**Ki-67**					
Positive	325	47	79.23%	0.58	0.52–0.64
Negative	104	251			

*ER* estrogen receptor, *PR* progesterone receptor, *HER2* human epidermal growth factor receptor 2, *ER/PR-negative* < 1%, *ER/PR-positive* ≥ 1%, *HER2-positive* IHC score 3 + or IHC 2 + /ISH-positive, *HER2-negative* IHC scores 0, 1 + , or 2 + /ISH-negative, *Ki-67-negative* < 20%, *Ki-67-positive* ≥ 20%

**Fig. 1:**
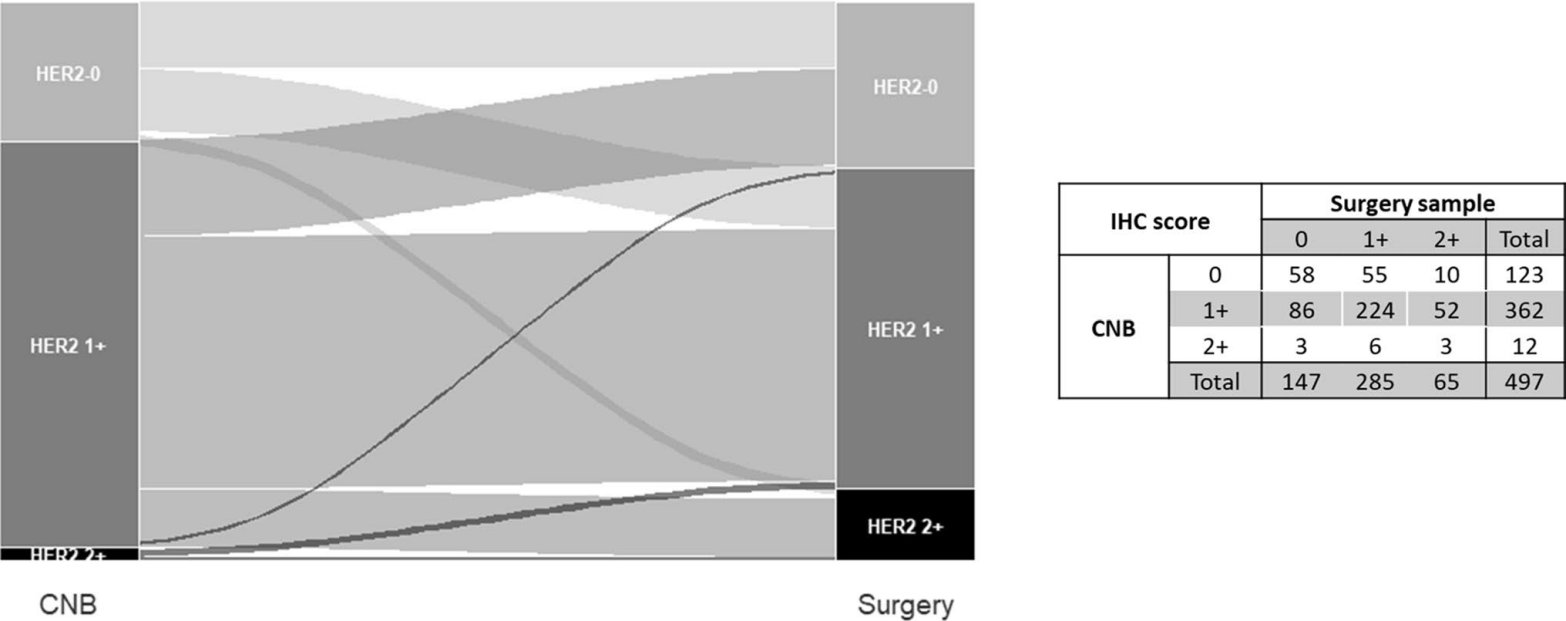
Changes of IHC scores from CNB to surgical specimens in HER2-negative breast cancer

**Fig. 2:**
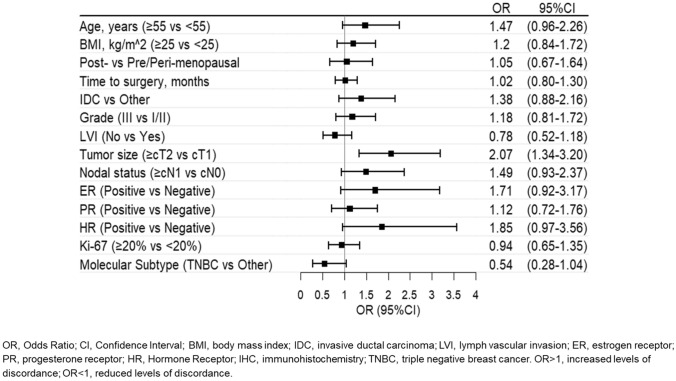
Potential determinants of discordance between CNB and surgical samples in HER2-negative breast cancer

Conversely, the number of discordant cases was considerable among HER2-negative cases (Fig. 1). While in CNB, 123 samples showed an IHC score of 0 and 374 had an IHC score of 1 + or 2 + /ISH-negative, in surgical specimens 147 samples were scored as IHC-0 and 350 as IHC score of 1 + or 2 + /ISH-negative, resulting in an observed agreement of 69% and a Kc of 0.22 (95%CI: 0.13–0.31), indicating only fair agreement. Among the 154 discrepant cases, 65 patients had a score of 0 on CNB that turned into 1 + or 2 + /ISH-negative on the surgical specimen, while 89 patients had a score of 1 + or 2 + /ISH-negative on CNB that turned into 0 on the surgical specimen. In the search for determinants of discordance (Fig. 2), tumor size emerged as the only significant clinico-pathological factor. Specifically, discordance between CNB and surgical specimens was twice more likely when the clinical tumor size was larger than 2 cm compared to smaller tumors (OR: 2.07; 95% CI: 1.34–3.20). The determinants of discordance were also assessed according to pathological characteristics and the results are reported in Supplementary Fig. 3.

## Discussion

Breast cancer oncologists rely on preoperative core needle biopsies for diagnostics and risk classification, which are essential for guiding therapy decisions. Several studies have compared CNB and surgical samples regarding ER, PR, HER2 immunostaining, demonstrating high agreement rates [14]. Our study reported a 98% agreement for ER- and 95% for HER2-positive cases, aligning with the literature, and confirming that treatment decisions and potential outcomes for patients based on CNB testing are accurate and reliable.

In addition to these consistent results, we present for the first time a comprehensive evaluation of HER2 in a large, prospectively maintained single-institution case series using CNBs and matched surgical samples in 776 treatment-naïve early breast cancer patients. HER2-low breast cancer, a recently identified category arising from the advent of antibody–drug conjugate (ADC) therapies, was numerically higher in CNB specimens compared to paired surgical samples. However, half of the HER2-0 tumors initially classified by CNB were reclassified as HER2 IHC score 1 + or 2 + /ISH-negative in surgical specimens, underscoring the importance of re-evaluating HER2 status in surgical samples to guide emerging anti-HER2 therapies. Conversely, it is desirable to establish a more accurate and objective method for categorizing HER2 as zero or low, as we have attempted through gene expression profiling and as other groups continue to explore with omics techniques.

Challenges in accurately determining HER2 status include issues with sample preparation, assay techniques, and intratumoral heterogeneity. The small size of CNB samples may not fully represent the tumor HER2 expression profile. In line with this, our data show that tumors larger than 2 cm are more likely to have HER2 discordance, highlighting significant variability in HER2 status within tumors and technical inconsistencies in testing methods. Consistent with other authors, we found that larger T size, increased stage, and nodal involvement—though to a lesser extent—reduced concordance between CNB and the surgical specimen, possibly because all are associated with increased heterogeneity of the primary tumor [15, 16].

While the retrospective nature of the study poses limitations—particularly regarding HER2 status evaluation, which may introduce diagnostic and selection biases due to historical focus on identifying positive cases rather than distinguishing negative categories—its strengths include a large, consecutive, mono-institutional cohort evaluated consistently by dedicated breast pathologists throughout the study period, providing valuable insights into the clinical and biological aspects of HER2-low status. Furthermore, unlike other studies that reported kappa statistics without consistently including a (1-α)% Confidence Interval (CI) [17] or properly handling ordinal data [15], our study provides unweighted kappa statistics for a two-level nominal scale with the corresponding CI, ensuring precise and reliable results.

In conclusion, our findings show a potential issue with CNB misdiagnosing HER2-negative breast cancer particularly in large tumors. This is important as CNB may be the only source of information for patients undergoing primary systemic therapy with novel therapies against breast cancer with low HER2 expression.

## Supplementary Information

Below is the link to the electronic supplementary material.

Supplementary file1 (PDF 178 KB)

## Declarations

### Data availability

Data are available upon reasonable request from the authors.
