# Supplementary material for: Reliability of core needle biopsy for HER2-low early-stage breast cancer
Source: Clin Transl Oncol. 2025 Mar 9;27(8):3340–5. doi: 10.1007/s12094-025-03877-2 (PMC12259780; doi:10.1007/s12094-025-03877-2)
Supplement: Supplementary file 1 — Supplementary file1 (PDF 178 KB) [file 12094_2025_3877_MOESM1_ESM.pdf]

Reliability of core needle biopsy for HER2-low early-stage breast cancer

Clinical and Translational Oncology

Chiara M. Ciniselli[1*], Paolo Verderio[1*], Valeria Duroni[1], Paolo Baili[2], Sara Pizzamiglio[1], Filippo G. de Braud[3,4], Secondo Folli[5], Catherine Depretto[6], Gianfranco Scaperrotta[6], Maria C. De Santis[5,7], Maria G. Carnevale[7], Cinzia De Marco[8], Andrea Vingiani[4,5,8], Giancarlo Pruneri[4,5,8] and Serena Di Cosimo[8].

[1]Bioinformatics and Biostatistics Unit, Department of Epidemiology and Data Science, Fondazione IRCCS Istituto Nazionale dei Tumori, 20133, Milan, Italy;

[2]Data Science Unit, Department of Epidemiology and Data Science, Fondazione IRCCS Istituto Nazionale dei Tumori, 20133, Milan, Italy;

[3]Department of Medical Oncology and Hematology, Fondazione IRCCS Istituto Nazionale dei Tumori, 20133, Milan, Italy;

[4]University of Milan, Italy;

[5]Breast Unit, Fondazione IRCCS Istituto Nazionale dei Tumori, 20133, Milan, Italy;

[6]Breast Imaging Unit, Fondazione IRCCS Istituto Nazionale dei Tumori, 20133, Milan, Italy;

[7]Radiation Oncology 1, Fondazione IRCCS Istituto Nazionale dei Tumori, 20133, Milan, Italy;

[8]Department of Advanced Diagnostics, Fondazione IRCCS Istituto Nazionale dei Tumori, 20133, Milan, Italy.

* These authors contributed equally to this work

Correspondence to:

Paolo Verderio, PhD

Bioinformatics and Biostatistics Unit,

Department of Epidemiology and Data Science,

Fondazione IRCCS Istituto Nazionale dei Tumori, Milan, 20133 Italy

e-mail: paolo.verderio@istitutotumori.mi.it

ORCID-ID: orcid.org/0000-0002-9231-1281

Supplementary Figure 1. Consort of study patient population selection

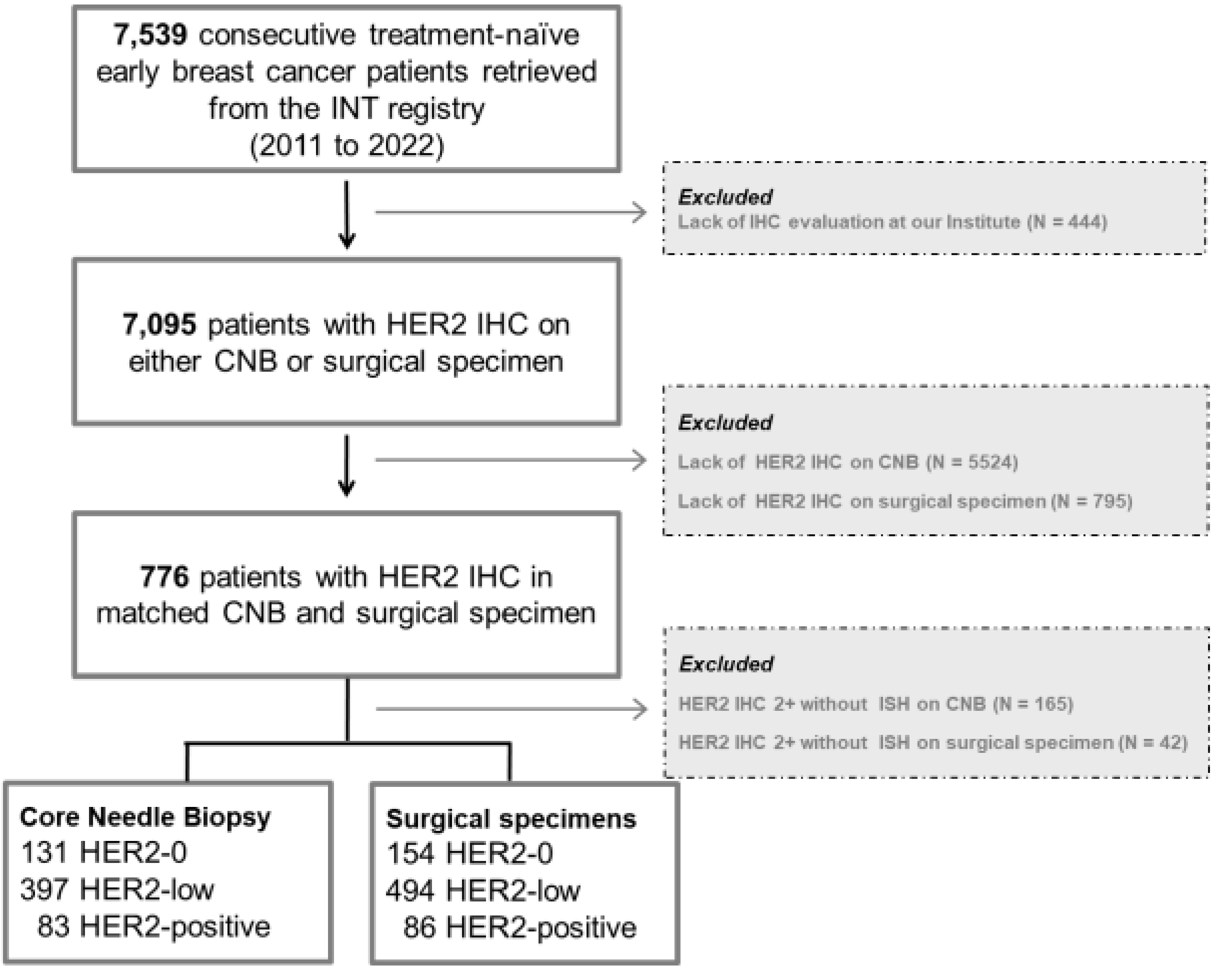

Supplementary Figure 2. Levels of concordance between CNB and surgical specimens in HER2-negative breast cancer over time

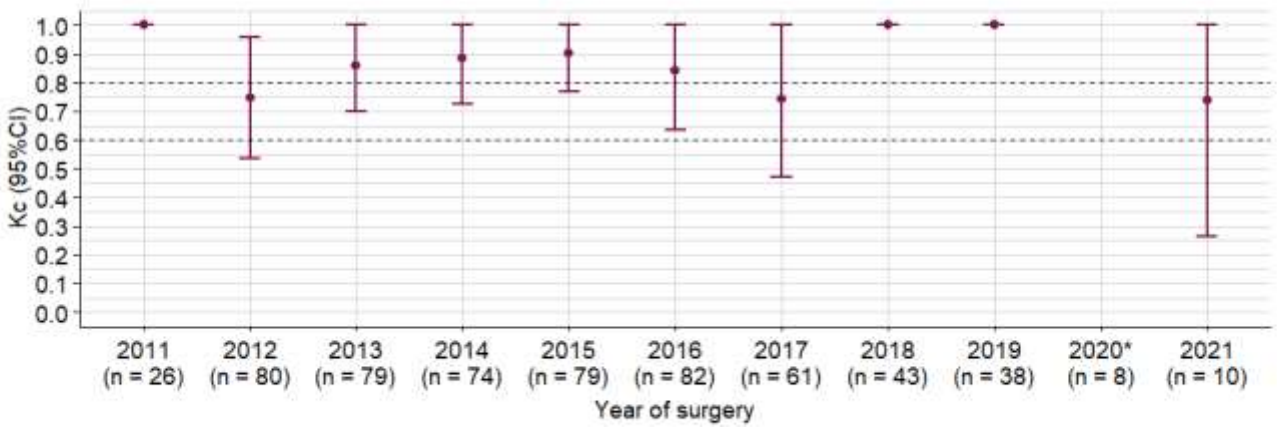

Unweighted kappa statistics (k_c) estimates together with the 95% Confidence Intervals (95%CI) over time according to the year of diagnosis. Dots indicate the k_c point estimates and the solid vertical lines extend to the lower and upper value of the 95% CI. The horizontal dashed lines delimit the ranges of Landis and Koch's classification criteria according to which the Choen kappa values is interpreted (i.e., 0.61 and 0.81 for a *moderate* and *almost perfect* agreement, respectively).

Supplementary Figure 3. Potential determinants of discordance between CNB and surgical samples in HER2-negative breast cancer by considering the pathological characteristics

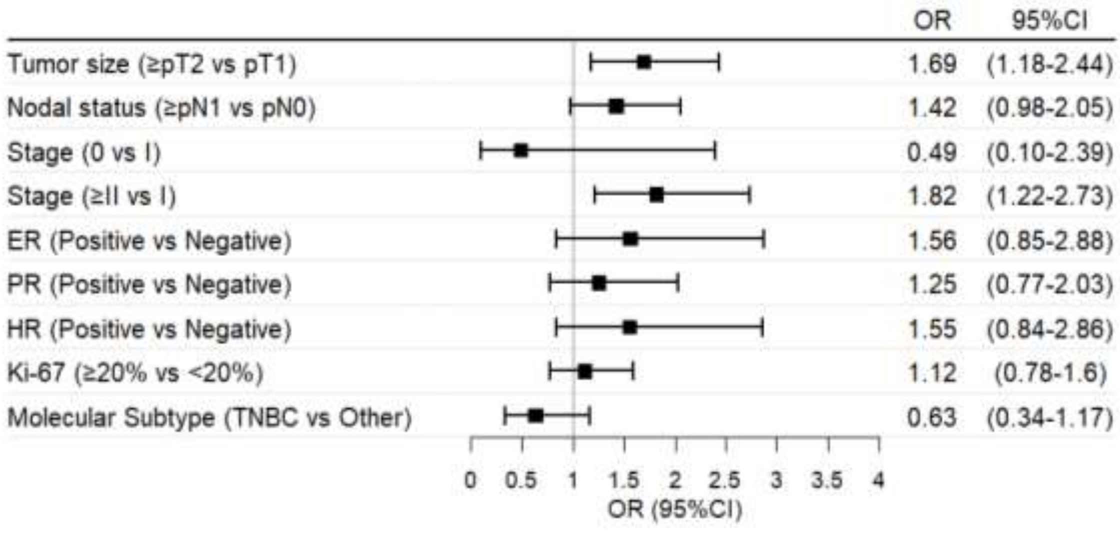

OR, Odds Ratio; CI, Confidence Interval; ER, estrogen receptor; PR, progesterone receptor; HR, Hormone Receptor; IHC, immunohistochemistry; TNBC, triple negative breast cancer. OR>1, increased levels of discordance; OR<1, reduced levels of discordance.
